# Does *Fusobacterium* in Colorectal Cancer Sites Originate From the Oral Cavity? A Pilot Study

**DOI:** 10.1002/cre2.70016

**Published:** 2024-11-03

**Authors:** Niels Plomp, Kristina Bertl, Marie‐Louise Lydrup, Klas Sjöberg, Hermie J. M. Harmsen, Andreas Stavropoulos

**Affiliations:** ^1^ Department of Medical Microbiology and Infection Prevention, University Medical Center Groningen University of Groningen Groningen The Netherlands; ^2^ Department of Periodontology, Dental Clinic, Faculty of Medicine Sigmund Freud University Vienna Vienna Austria; ^3^ Department of Periodontology Blekinge Hospital Karlskrona Sweden; ^4^ Department of Surgery Skåne University Hospital and Lund University Lund Sweden; ^5^ Department of Clinical Sciences Lund University Malmö Sweden; ^6^ Department of Gastroenterology and Nutrition Skåne University Hospital Malmö Sweden; ^7^ Periodontology, Faculty of Odontology University of Malmö Malmö Sweden; ^8^ Division of Conservative Dentistry and Periodontology, University Clinic of Dentistry Medical University of Vienna Vienna Austria; ^9^ Department of Periodontology, School of Dental Medicine University of Bern Bern Switzerland

**Keywords:** 16S rRNA sequencing, colorectal cancer, culturomics, *Fusobacterium*, PCR

## Abstract

**Objectives:**

*Fusobacterium* can contribute to oral diseases, but also pose as a systemic risk factor. This genus, and especially *F. nucleatum*, can be found in colorectal cancer (CRC) tissue and is involved in multiple aspects of this type of cancer. Previous studies indicated a possible oral origin of these bacteria; however, stronger evidence is needed to reach a definitive conclusion. This pilot study aimed to establish a method to successfully compare, at the strain level, fusobacteria from the oral cavity and CRC resection material for future cohort studies of CRC patients.

**Material and Methods:**

In a first cohort of eight periodontitis patients, gingival crevicular fluid and saliva were collected. *Fusobacterium* was isolated on two different media. In a second cohort, saliva and CRC resection material were collected from ten CRC patients. These samples were used for screening of *Fusobacterium* with culturing, 16S rRNA gene profiling and a PCR‐based approach.

**Results:**

In the first cohort, different *Fusobacterium* species were identified in GCF and saliva samples. However, as the total yield of *Fusobacterium* seemed slightly higher in saliva samples, it was therefore preferred for subsequent sample collection. Thus, in the second cohort, patient‐matched saliva and CRC resection material were screened for *Fusobacterium* and this showed that nine patients were culture‐positive in the saliva samples; however, no *Fusobacterium* could be isolated from the resection material. On the other hand, 16S rRNA gene profiling of the resection material indicated that eight CRC patients were positive for *Fusobacterium*. All eight of these patients carried *Fusobacterium* in their saliva, indicated by both marker gene PCR and culture‐based screening.

**Conclusions:**

These pilot results are compatible with data from previous studies, indicating a possible link between oral and CRC‐associated *Fusobacterium*, and a more in‐depth analysis of specific strains and their characteristics in a larger cohort is justified.

**Trial Registration:**

The protocol was registered at clinicaltrials.gov (NCT05945082).

## 1 Introduction

Colorectal cancer (CRC) ranks fifth in terms of global incidence and fourth in terms of the mortality rate compared to other cancer types (Sung et al. 2021). Genomic studies have provided the earliest evidence that bacteria are prevalent in CRC tumor tissue; especially *Fusobacterium nucleatum* has been associated with CRC, although other fusobacteria also reside in the colon (Castellarin et al. 2012; Kostic et al. 2012; Richardson et al. 2020). Later reports indicated that this bacterium is involved in multiple aspects of CRC development and progression (Yu, Kim, and Park 2020; Yang et al. 2017; Rubinstein et al. 2019; Kostic et al. 2013; Gur et al. 2019; Casasanta et al. 2020; Chen et al. 2020; Zhang et al. 2019; Yu et al. 2017). The tumor progression is accelerated by *Fusobacterium*‐mediated activation of the epidermal growth factor receptor (EGFR) signaling pathways, which stimulates the epithelial–mesenchymal transition of tumor cells (Yu, Kim, and Park 2020), and *Fusobacterium* also accelerates proliferation of CRC cells by Toll‐like receptor 4 (TLR4)‐activated NF‐κB signaling (Yang et al. 2017) and activation of the Wnt/β‐catenin pathway (Rubinstein et al. 2019). Additionally, *Fusobacterium* selectively recruits tumor‐infiltrating myeloid cells that generate a pro‐inflammatory microenvironment (Kostic et al. 2013) and prevents killing of CRC cells by inhibition of natural killer cells and tumor‐infiltrating T‐cells (Gur et al. 2019). *Fusobacterium* also drives metastasis of CRC cells (Casasanta et al. 2020; Chen et al. 2020). Finally, *Fusobacterium* makes CRC cells more resistant to chemotherapy due to modulation of the host autophagy and active inhibition of apoptosis, both through activation of the TLR4/NF‐κB signaling pathway (Zhang et al. 2019; Yu et al. 2017). Taking all this into account, it is not surprising that the prevalence and abundance of *Fusobacterium* in CRC tumor tissue have been associated with an overall poorer clinical outcome (Mima et al. 2016); nevertheless, controversial results (i.e., better CRC treatment outcomes) have also been reported (Alexander et al. 2023).

*Fusobacterium* is a genus of obligate anaerobic and gram‐negative bacteria that tend to form filamentous rods with tapered ends. It is a diverse genus, consisting of several species and subspecies, of which most are commonly found in the oral cavity and are among the highest abundant members of the oral microbiota (Socransky et al. 1998; Motoc et al. 2023). Although they are commensal bacteria, fusobacteria are associated with periodontitis, being crucial in the formation of subgingival biofilm (Socransky et al. 1998; Zijnge et al. 2010, 2012; Ximénez‐Fyvie, Haffajee, and Socransky 2000).

Poor oral health is an important global problem, especially in adults (Peres et al. 2019). In 2019, over 3.5 billion people were suffering from an oral disorder according to the Global Burden of Disease study, corresponding to approximately 45% of the global population (Global Burden of Disease Collaborative Network 2020). Although oral diseases, such as dental caries and periodontitis, can have a major impact within the oral cavity (e.g., tooth loss), they also represent a substantial systemic risk factor. Specifically, periodontitis, a chronic inflammatory disease of the tooth‐supporting structures caused by dysbiosis in a susceptible host (Lamont, Koo, and Hajishengallis 2018), has been linked to the development, progression, and severity of several systemic, mostly inflammation‐driven diseases. This link between periodontitis and systemic disorders is primarily based on two pathways, that is, via dissemination of oral or periodontal pathogens and due to the chronic inflammatory burden (Hajishengallis 2022). Periodontitis has been associated, among other diseases, with diabetes mellitus (Borgnakke 2019; Graziani et al. 2018), cardiovascular diseases (Sanz et al. 2020), autoimmune disease (Rodríguez‐Lozano et al. 2019; Hajishengallis and Chavakis 2021), inflammatory bowel diseases (Bertl et al. 2022; Madsen et al. 2023; Domokos et al. 2022), and various cancers, including CRC (Fu et al. 2022; Li et al. 2021).

Interestingly, recent research based on only three positive patient‐matched samples has hinted that CRC‐associated fusobacteria may originate from the oral cavity, most likely translocating via the hematogenous route and thus highlighting the importance of oral health (Abed et al. 2020). To overcome the difficulties in the isolation of fusobacteria from CRC material, more recently, whole‐genome sequencing and a PCR product of the hypervariable clustered regularly interspaced short palindromic repeats (CRISPR)‐associated regions were used (Shimomura et al. 2023). In line with previous results, these data also indicated a possible oral origin of CRC‐associated fusobacteria; however, only a small sample size was used for whole‐genome sequencing (i.e., five patient‐matched samples), whereas limited marker genes instead of the whole genome were analyzed for the remaining samples that were not sequenced.

Therefore, the aim of this pilot study was to establish a method to successfully compare fusobacteria at the strain level from the oral cavity and CRC resection material, as it is expected that this genus is present in both locations and CRC‐associated *Fusobacterium* might have an oral origin. To this end, after comparing different oral sampling methods, patient‐matched saliva samples and CRC resection material were used to detect different *Fusobacterium* species with either a culture‐based approach, 16S rRNA gene profiling, or marker gene PCR.

## 2 Methods

### 2.1 Patient Population

Two different patient populations were included. The first population (*n* = 8; “periodontitis patients”) included patients diagnosed with periodontitis Stage 3 or 4 (Tonetti, Greenwell, and Kornman 2018). These patients were visiting the Department of Periodontology (Faculty of Odontology, University of Malmö, Sweden) for regular supportive periodontal care and had at least one tooth with a probing pocket depth of ≥ 5 mm. The reason why periodontitis patients were chosen for this first population was to collect oral samples from a patient group with high chances of harboring a sufficient amount of *F. nucleatum* to simplify the decision process on the appropriate oral sampling method, but not to relate the results to the periodontal status of these patients. The second population (*n* = 10; “CRC patients”) included patients who were diagnosed with CRC and scheduled for surgical treatment at the Skåne University Hospital (Malmö, Sweden). Gender, age, and smoking status were recorded for both populations, whereas cancer diagnosis and localization and intake of antibiotics in the preceding 3 months were additionally recorded in the second population (Table 1). The protocol was approved by the regional ethical review board (Dnr. 2016/469) and registered at clinicaltrials.gov (NCT05945082).

**Table 1:** Characteristics of the CRC patients (*n* = 10).

Patient	Gender Smoking	Age BMI	Diagnosis Type	Localization	Intake of systemic antibiotics (in the preceding 3 months)	Comorbidities
9	m Never	67.4 20.4	Cancer Adenocarcinoma	Left, rectal	Yes	Chronic lymphocytic leukemia
10	m Never	86.4 25.8	Cancer Adenocarcinoma	Left	Yes	Hypertension
11	f Never	90.9 28.2	Not cancer Adenoma, low‐grade dysplasia	Right	Yes	Hypertension, pacemaker, pulmonary embolism
12	m Never	65.1 26.4	Cancer Adenocarcinoma	Left	No	Hypertension
13	m Former	86.7 25.8	Cancer Adenocarcinoma	Left	No	Atrial flutter, cerebral vascular insult, acute myocardial infarction, macroglobulinemia
14	m Current	78.5 32.4	Cancer Adenocarcinoma	Left, rectal	No	Atrial flutter, hypertension, chronic obstructive lung disease, benign prostata hyperplasia
15	f Never	76.7 20.0	Cancer Adenocarcinoma	Left, rectal	No	Breast cancer, hypertension
16	m Former	68.0 26.6	Cancer Adenocarcinoma	Right, appendix	Yes	Goiter, psoriasis
17^a^	f Former	80.9 23.2	Cancer Adenocarcinoma	Left	Yes	Hypertension, deep vein thrombosis
18	f Never	77.5 21.8	Cancer Adenocarcinoma	Left	No	Hypertension, diabetes mellitus, atrial flutter

Abbreviations: BMI, body mass index score; f, female; m, male.

a Re‐treatment; first surgery for cancer 12 years ago.

### 2.2 Sampling of Gingival Crevicular Fluid (GCF) and Saliva in the Periodontitis Patients

In each patient, one tooth with a probing pocket depth of ≥ 5 mm was chosen randomly for subgingival microbiological sampling by collecting GCF with paper points. The selected tooth was protected from contamination with saliva and supragingival plaque was carefully removed. One sterile paper point (ISO 55) was inserted into the bottom of the pocket and kept in place for 30 s. This process was repeated at the same sampling site three times, that is, in total, four paper points were collected per sampling site. All paper points of the same sampling site were pooled and stored in 1 mL of eSwab Amies medium (Copan Group, Brescia, Italy) mixed with 3 mL of 20% glycerol at −80°C until analysis. For saliva sampling, a regular‐sized, pre‐packed FLOQSwab (Copan Group) was used to collect unstimulated, whole saliva. Although for the first two patients the saliva was collected only from the back of the tongue, for the next six patients, the saliva was collected from the cheeks, vestibulum, the floor of the mouth, the back of the tongue, and the palate to ensure that a sufficient amount of saliva was obtained. After collection, the swab was stored in 1 mL of eSwab Amies medium mixed with 3 mL of 20% glycerol at −80°C until analysis.

### 2.3 Sampling of Saliva and Resection Material in the CRC Patients

The saliva sampling in the CRC patients was performed with a swab from the cheeks, vestibulum, the floor of the mouth, the back of the tongue, and the palate, which was stored in 1 mL of eSwab Amies medium with 20% glycerol at −80°C until analysis. All saliva collections were performed maximum 24 h before surgical CRC treatment. After surgical resection of the colorectal tumor, one biopsy of approximately 3 × 3 × 3 mm from the tumor site was collected from each patient and stored in the same medium and temperature.

### 2.4 Sample Preparation Before Inoculation of Bacterial Media

Before inoculation of the media, CRC resection material was cut into smaller pieces and 0.25 g was homogenized in 200 µL of Amies medium (Copan Group) with 0.1 g of zirconium beads (0.55 mm; Biospec products, Bartlesville, USA) and one glass bead (3 mm, Biospec products) in a homogenizer (Precellys 24, Bertin Technology, Montigny‐le‐Bretonneux, France) at three cycles of 4500 RPM for 45 s with intervals of 45 s. After this, the samples were centrifuged at 3000 RPM for 1 min and the supernatant was used for bacterial isolation. All saliva samples were homogenized by vortexing before inoculation.

### 2.5 Isolation of *Fusobacterium* Species From GCF and Saliva Samples and CRC Resection Material

For the isolation of *Fusobacterium* from all samples, Brucella blood agar (Mediaproducts BV, Groningen, the Netherlands) and *Fusobacterium*‐specific medium (Fastidious anaerobe agar containing 3 mg/L josamycine, 1 mg/L norfloxacin, 4 mg/L vancomycin, and 5% defibrinated horse blood; Mediaproducts BV) were used and reduced overnight before the experiments. Fifty microliters of either the GCF or saliva samples or the CRC resection lysates was streaked into three segments per agar medium. After inoculation, the plates were incubated for 2 days under anaerobic conditions (10% H_2_, 10% CO_2_, and 80% N_2_) and at 37°C in a Whitley A35 Workstation (Don Whitley Scientific Ltd, Bingley, UK) and pure colonies were obtained using a classical pure culture technique that involved at least three rounds of streaking until single colonies were obtained, whose preliminary purity was assessed using Gram staining afterward.

### 2.6 Bacterial Identification and Storage

The identity of the bacterial isolates was assessed by matrix‐assisted laser desorption/ionization coupled to a time‐of‐flight mass spectrometer (MALDI‐TOF MS; Biotyper Microflex, Bruker Daltonics, Billerica, USA; database version 11) as described previously (Veloo et al. 2016). In short, single colonies were spotted on a polished stainless‐steel MALDI target. Air‐dried spots were covered with 1 µL of 2‐Cyano‐3‐(4‐hydroxyphenyl) acrylic acid matrix (10 mg/mL) and air‐dried again. Subsequently, the spotted isolates were analyzed by summing 240 measured shots, using the Biotyper Microflex (Veloo et al. 2016; Almuzara et al. 2016). Identification of the isolates was considered reliable to the species level if results had a log‐score of 2.0 or higher, between 1.7 and 1.99 for genus identification, and not reliable if lower than 1.7, as recommended by the manufacturer (Bruker Daltonics). All isolates that were reliably identified as *Fusobacterium* were stored at −80°C in a 1 mL mix of Brain–Heart infusion broth (Oxoid Ltd, Hampshire, UK) and 85% (v/v) glycerol at a ratio of 1:4 until further use.

### 2.7 Next‐Generation 16S rRNA Gene Amplicon Sequencing of CRC Resection Material

DNA from 0.25 g of the CRC resection material was extracted using a repeated bead‐beating method as described previously (de Goffau et al. 2013). The sequencing of the samples was performed according to previously described methods (Heida et al. 2016). In short, amplification was performed with PCR using modified 314 F and 806 R primers, targeting the V3–V4 region of the 16S rRNA gene. The samples were barcoded with six nucleotides added to the reverse primer. An in‐house Illumina MiSeq was used to sequence the samples on a 2 × 300 bp cartridge (Illumina Inc., San Diego, USA). Afterward, the reads were joined, quality control was performed (1% error rate), and the primers were cut using VSEARCH (Rognes et al. 2016). The denoising of the reads, chimeric read removal, singleton removal, and dereplication were performed using both VSEARCH and USEARCH (Rognes et al. 2016; Edgar 2010). The amplicon sequence variants (ASVs) were annotated using the Ribosomal Database Project (RDP) classifier based on the RDP set 18 (Cole et al. 2014; Wang et al. 2007). The relative abundance of the ASVs was calculated as the read count of each ASV divided by the total read count of the mapped reads after rarefaction to the same depth. The ASVs that were annotated as *Fusobacterium* were compared with reference 16S rRNA gene sequences of known species within the genus. All reference sequences were obtained from NCBI. The *Fusobacterium* ASVs and the reference sequences were aligned using MUSCLE (Edgar 2004). The determination of the phylogenetic relationship was based on the nucleotide substitution rates after elimination of all positions with gaps or missing data. The data were visualized with a phylogenetic tree that was constructed based on evolutionary distances of the maximum likelihood method (Tamura–Nei model) (Felsenstein 1981; Tamura and Nei 1993) using Mega software (version X) (Kumar et al. 2018). The confidence in topology of the tree was estimated with bootstrap values, which were calculated based on 1000 sampling replications (Felsenstein 1985).

### 2.8 PCR Amplification of a *Fusobacterium*‐Specific Conserved 16S rRNA Gene Region From Saliva Samples

The DNA from 0.25 g of the saliva samples was extracted using the same method, but the isopropanol in the precipitation step was omitted. To detect the presence of *Fusobacterium*, a genus‐specific conserved region of the 16S rRNA gene was amplified using the previously described PCR primers FUSO1 (forward primer: 5′‐GAG AGA GCT TTG CGT CC‐3′) and FUSO2 (reverse primer: 5′‐TGG GCG CTG AGG TTC GAC‐3′) (Nagano et al. 2007). Each PCR reaction consisted of 50% v/v 2X Phire Hot Start II PCR Master Mix (Thermo Scientific, Waltham, USA), 0.2 µM of each primer, and 37.5 ng of DNA dissolved in nuclease‐free water, resulting in an end volume of 25 µL. A program was run consisting of 1x denaturation at 98°C for 30 s, followed by 35 cycles of denaturation (98°C, 10 s), annealing (60°C, 10 s), and extension (72°C, 15 s), ending after these cycles with a final step of incubation at 72°C for 2 min. The final PCR products were separated with gel electrophoresis on a 1% agarose gel in 1x TBE buffer. All samples and the DNA ladder (FastRuler Low Range DNA Ladder, Thermo Scientific) were loaded with 6x MassRuler DNA Loading Dye (Thermo Scientific) and Midori Green advance DNA stain (Nippon Genetics). The loaded gel was run at 150 V for 30 min. Afterward, the DNA was visualized using a gel imaging system (Gel Doc EZ imager, BioRad, Hercules, USA).

## 3 Results

### 3.1 Mode of Sampling From the Oral Cavity Yielded Comparable Number of Different Cultured *Fusobacterium* Species

**Figure 1:**
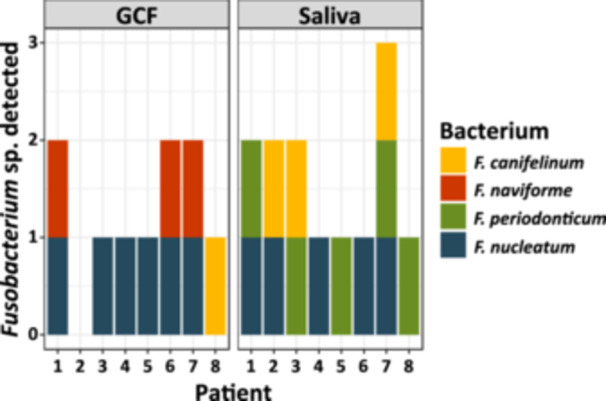
The stacked bar plots show the presence of cultured *Fusobacterium* species from GCF samples (on the left) and saliva samples (on the right) in eight periodontitis patients. The different patients are shown on the *X*‐axis and the *Y*‐axis indicates the number of different identified *Fusobacterium* species within each sample. Species identification by MALDI‐TOF MS is indicated by different colors, with *F. canifelinum* in yellow*, F. naviforme* in red, *F. periodonticum* in green, and *Fusobacterium nucleatum* in blue.

Among the periodontitis patients, there were three males and five females, aged between 34 and 72 years, two never smokers, two former smokers, and four current smokers. Figure 1 shows that GCF sampling resulted in the isolation of *F. nucleatum* in six samples, whereas five saliva samples were positive for this bacterium; except for patient two, the positive saliva samples were in agreement with the GCF samples. Interestingly, cultivation from GCF samples resulted in the isolation of *F. naviforme*, which could not be isolated from saliva samples. On the other hand, *F. periodonticum* was exclusively cultured from saliva samples, whereas GCF samples were culture‐negative for this species. Although the mode of sampling resulted in different *Fusobacterium* species, the total yield of this genus seemed slightly higher in saliva samples. Due to this and as collection of saliva with swabs from the oral cavity is easier and less prone to contamination, it was decided to move forward with this sampling method for further collection.

### 3.2 Targeted Culturing Showed That Different *Fusobacterium* Species Were Highly Present in the Oral Cavity of CRC Patients, But Could Not be Cultured From CRC Resection Material

**Figure 2:**
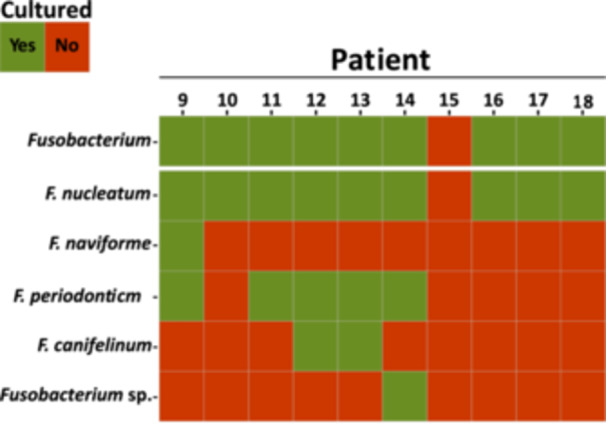
Heat map representation of the culture‐based screening for *Fusobacterium* in the CRC patients, represented on the *X*‐axis with their patient numbers 9−18. The *Y*‐axis shows the cultured presence (green) or absence (red) of this genus in general (top row), specific species (middle rows), and isolates that could only be annotated to the genus level (bottom row).

All CRC patients, except Patient 15, were culture‐positive for the genus *Fusobacterium* in the saliva samples. The species *F. nucleatum* was isolated in all culture‐positive patients (Figure 2). In 55.6% of all culture‐positive patients, *F. periodonticum* was found to be the second most abundant species within this genus. On the other hand, only one patient was culture‐positive for *F. naviforme*, a species that is also associated with infections in gingival tissue (Bao et al. 2020), and one patient was culture‐positive for other *Fusobacterium* species that could not be identified from our MALDI‐TOF MS database (Figure 2). Isolation of *Fusobacterium* from the CRC resection material by culturing was not successful (data not shown).

### 3.3 16S rRNA Gene Amplicon Sequencing Revealed Presence of *Fusobacterium* in CRC Resection Material and Phylogenetic Analysis of the ASVs Indicated the Presence of Multiple Species in the Tumor Site

**Figure 3:**
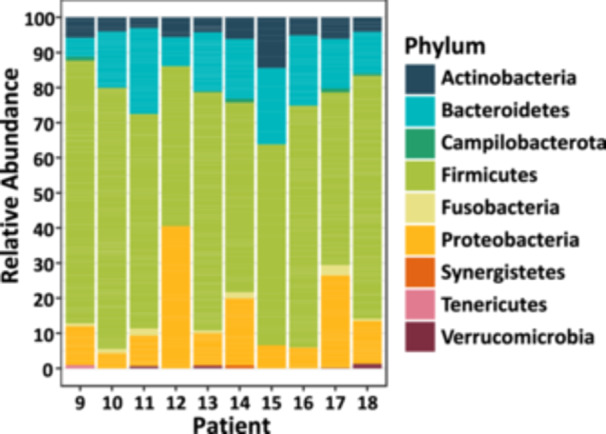
The relative abundances of bacterial phyla in the CRC resection material are represented in a stacked bar plot. The 16S rRNA gene profiling shows that eight CRC patients are positive for the phylum *Fusobacteria*, which where all annotated as the genus *Fusobacterium* (Table S1).

As *Fusobacterium* was not successfully isolated from the CRC resection material by culturing, the bacterial communities were characterized based on 16S rRNA gene amplicon sequencing. Most bacterial genera in the samples belonged to the *Firmicutes* phylum (Figure 3; 45.6%−74.8%). This phylum was previously reported to be among the most abundant in the human gut microbiota (Gacesa et al. 2022; Zhernakova et al. 2016; Turnbaugh et al. 2007). Interestingly, most of the samples were colonized with the phylum *Fusobacteria,* of which *Fusobacterium* is the only represented genus in our data set (Table S1). Eight out of the ten samples were positive for this genus, except for patients 12 and 15 (Figure 3). The relative abundance of this genus ranged between 0.1% (Patient 16) and 2.9% (Patient 17) in the patients who were positive.

**Figure 4:**
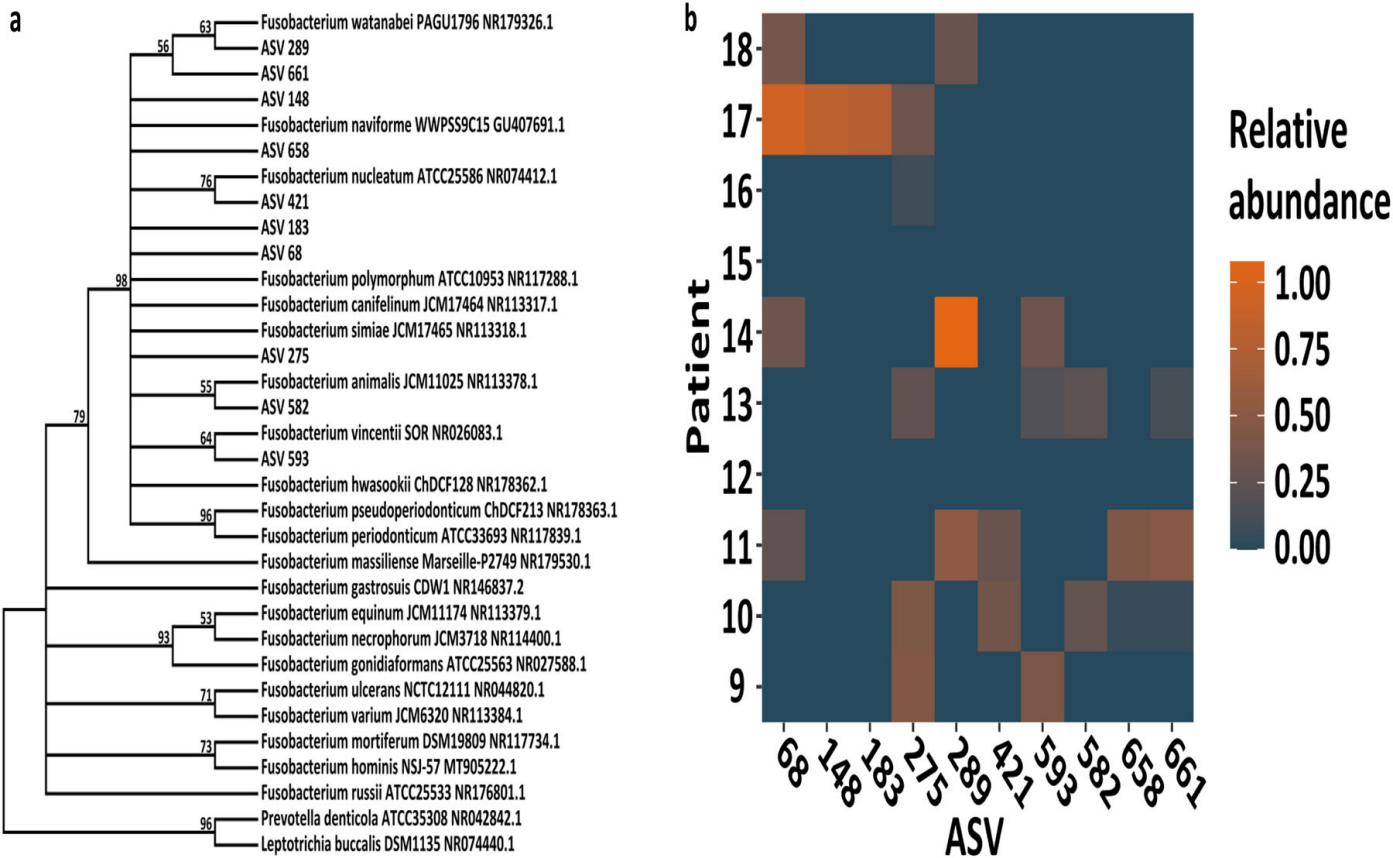
(a) The 16S rRNA gene ASVs are clustered together with known references of this genus by the maximum likelihood method with the Tamura–Nei model using 401 nucleotides. All positions containing gaps and missing data were eliminated. The bootstrap consensus tree from 1000 replicates is shown. The percentage of replicate trees in which the associated taxa clustered together in the bootstrap test is shown next to the branches, and branches with less than 50% replication are collapsed. The strain names of the references and accession numbers are given behind the bacterial names. (b) Heat map representation of the relative abundances of the fusobacterial ASVs in the CRC resection material. The patient numbers are displayed on the *Y*‐axis and the different ASVs are displayed on the *X*‐axis. The relative abundance is indicated by different colors, ranging from 0 in blue to 1 in orange.

Most saliva samples were culture‐positive with certain species of *Fusobacterium*; therefore, evolutionary analysis was performed on the ASVs that were found in the resection material together with reference sequences of all representative *Fusobacterium* members to gain more insight if this is also true for this type of material. This clustering analysis showed that all fusobacteria of the CRC resection material clustered into two distinct groups and every ASV fell into the same group as *F. nucleatum* and other closely related (sub)species that were previously identified by culturing of the saliva samples (Figure 4a). As shown in Figure 4b, at least one ASV annotated to this genus could be found in all positive patients (i.e., in all patients, except for Patients 12 and 15) and this could even go up to five different ASVs present in two of these patients (i.e., Patients 10 and 11). Most patients carried ASV275, which could be identified in five different patients. ASV numbers 289, 68, 148, and 183 had the highest relative abundance in individual patients. In addition, ASV numbers 289, 68, and 275 had the highest total relative abundance in this cohort.

### 3.4 All *Fusobacterium*‐Positive CRC Patients Had PCR‐Positive Saliva Samples, Possibly Linking Oral and CRC‐Associated *Fusobacterium*

The presence of the genomic content of *Fusobacterium,* despite the negative culture results in the CRC resection material, led to a reanalysis of the saliva samples. Unfortunately, the obtained DNA was not suitable for 16S rRNA profiling; however, it could still be used for marker gene PCR. The gel electrophoresis (Figure S1) of a genus‐specific marker showed that the saliva samples of all patients, except for Patient 15, contained DNA of *Fusobacterium*, which was also in line with the culture results of these samples.

**Figure 5:**
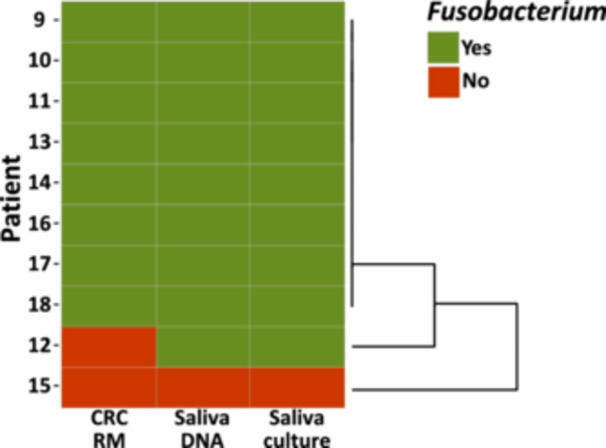
Heat map representation of the presence (green) or absence (red) of *Fusobacterium* by 16S rRNA profiling in CRC resection material (CRC RM), marker gene PCR from saliva (saliva DNA), and the culture results from saliva samples (saliva culture). This overview shows that eight patients were positive in both sites, one patient carried only oral *Fusobacterium*, and in one patient, *Fusobacterium* could not be detected in either of the sites. These three groups are also shown by clustering on the right.

Comparison of the presence of *Fusobacterium* genomic content between patient‐matched saliva samples and CRC resection material (Figure 5) revealed that this pilot group of patients clustered into three subgroups. Eight out of ten patients carried these bacteria both in their oral cavity and in their tumoral site. One patient carried *Fusobacterium* in the oral cavity, but the presence of this genus was not confirmed in the CRC resection material. Finally, one patient was not a carrier of this genus either in the oral cavity or in the tumor site.

## 4 Discussion

Culturing methods and genomic analysis were carried out in this study, which showed that *Fusobacterium* was present in both patient oral samples and CRC resection material. On the basis of saliva and CRC resection material collected from ten CRC patients, *Fusobacterium* could be identified with culturing in the saliva samples of nine patients, but no *Fusobacterium* could be isolated from the CRC resection material. On the other hand, 16S rRNA gene profiling of the resection material indicated that eight CRC patients were indeed positive for *Fusobacterium*; all eight of these patients carried *Fusobacterium* also in their saliva.

These findings are in accordance with previous studies, that *Fusobacterium* is prevalent in CRC tumor tissue; however, the origin of these fusobacteria is still unclear. Early evidence suggests that these bacteria might originate from the oral cavity (Castellarin et al. 2012; Kostic et al. 2012; Richardson et al. 2020). Still, more evidence is needed to conclude that CRC‐associated fusobacteria indeed have an oral origin. In this study, *Fusobacterium* was identified in CRC resection material of several patients, along with other pathogenic and opportunistic genera, such as *Campylobacter*, *Burkholderia*, and *Serratia*. Yet, *Fusobacterium* has already been shown to be involved in a multitude of steps in CRC development and progression (Yu, Kim, and Park 2020; Yang et al. 2017; Rubinstein et al. 2019; Kostic et al. 2013; Gur et al. 2019; Casasanta et al. 2020; Chen et al. 2020; Yu et al. 2017).

To investigate the possible oral origin of fusobacteria in CRC tumor tissue, oral samples were also collected herein. First, different oral sampling methods were studied in a small group of volunteers. In general, there are various oral sampling methods available with relevant differences among each other and the method should be chosen according to the design and aim of a specific research question (Lu et al. 2022). Herein, it was decided to compare GCF with saliva samples as the main aim was to collect *Fusobacterium*. It has been shown previously that supragingival plaque samples, which would have been a third option, are often more heterogeneous in their constitution compared to subgingival ones, whereas subgingival plaque samples rather consistently harbor *Fusobacterium* (Zijnge et al. 2010). The present analysis indicated that the culturable species diversity was comparable between saliva and GCF samples. This is in line with previous results from the literature, showing that bacterial compositions with these two sampling methods are comparable (Haririan et al. 2014). However, the two methods differ in their applicability, as GCF sampling is more prone to contamination and thus less suitable for collection in a hospital setting. Taking all this into consideration, the saliva sampling method was used in the cohort of CRC patients. Initially, the objective was to culture and isolate *Fusobacterium* from oral samples and CRC resection material, after which whole‐genome sequencing would be performed on all isolated strains. The oral samples were almost all culture‐positive for *Fusobacterium*, as expected, as *Fusobacterium* is one of the most prominent oral microbes (Socransky et al. 1998; Motoc et al. 2023). The presence of *Fusobacterium* was also confirmed with genus‐specific marker gene PCR and mirrored the culturing results, showing that a given patient either carried or did not carry this genus. This gave the confidence that the protocol for culture‐based screening in this study was appropriate for this type of sample, as the bacterial DNA load corresponded to the presence of viable bacteria. Viable and multiplying bacteria are essential for culture‐based screening, which is both the biggest advantage and disadvantage. The advantage is that only bacteria that will grow are detected, and pure colonies can be used subsequently to obtain high‐resolution genome sequences. At the same time, the biggest drawback is that bacteria might lose viability before sampling, as live microbial cell loads may fluctuate in orders of magnitude across participants or time points (Marotz et al. 2021) or during every step of the protocol, or they may require other specific growth conditions than those implemented. This drawback was found with the CRC resection material, where no fusobacteria could be isolated from the patient‐matched samples using our protocol. Previously, however, we had successfully isolated *Fusobacterium* during optimization with CRC resection material of four patients, of which one was culture‐positive (data not shown). Isolation of these bacteria from this material was already previously described to be difficult, ascribed to the pre‐procedural antibiotics that patients receive (Abed et al. 2020). For this reason, antibiotic usage in the 3 preceding months was recorded in our cohort; not all patients had received antibiotics but were still culture‐negative for *Fusobacterium*. To tackle the drawbacks of a culture‐based approach, DNA was isolated to perform 16S rRNA amplicon sequencing. Interestingly, 16S rRNA profiling of the resection material revealed that almost all patients who carried oral fusobacteria also had CRC‐associated bacteria of this genus, even though some patients received antibiotics within the 3 preceding months, which was previously suggested to reduce the presence of *Fusobacterium*. However, the resolution of 16S rRNA profiling depends on the amplicon size and, in general, the bacteria could only be identified with confidence to the genus level due to the short amplicon size of 407 base pairs after trimming. Still, clustering of the fusobacterial ASVs after alignment with reference species and with a 95% cut‐off value after bootstrapping suggested that all CRC‐associated fusobacteria belong to the cluster of *F. nucleatum* and closely related species, which could be cultured from the oral samples as well. Unfortunately, the quality of the isolated salivary DNA was too poor to perform 16S rRNA gene profiling, possibly due to the relatively low bacterial biomass compared to host DNA and the fact that no bacterial DNA enrichment protocol was implemented, which was previously described to improve sequence quality (Marotz et al. 2021, 2018).

Altogether, these results underline an interesting link between oral and CRC‐associated fusobacteria. However, more in‐depth analysis is needed to conclude that specific strains are indeed shared between the two locations. In addition, strain‐specific genes that are enriched in CRC‐associated strains could be analyzed, as previous reports indicated that there is a significant difference in gene composition, for instance, regarding virulence factors or antibiotic resistance (Ma et al. 2023). In particular, the latter is of interest, because antibiotic treatment of patients might ameliorate the clinical outcome by reduction of *Fusobacterium* load, CRC cell proliferation, and overall tumor growth as shown in mice (Bullman et al. 2017). This in‐depth analysis can be carried out with high‐resolution metagenomic sequencing in combination with depletion of host DNA to increase the resolution of bacterial reads (Marotz et al. 2018). Finally, from a clinical point of view, it will be interesting to assess in future studies with a larger sample size whether patient‐related parameters (e.g., smoking status, intake of antibiotics, comorbidities, etc.), the oral health status per se (e.g., periodontally healthy vs. diseased, severity and extent of periodontal disease, etc.), and/or the oral sampling method (e.g., subgingival vs. supragingival vs. saliva) have an impact on the colonization and/or the detection rate of oral fusobacteria at the CRC site.

## 5 Conclusion

On the basis of culturing methods and genomic analysis performed in this study, it was shown that *Fusobacterium* was present in both oral samples and CRC resection material; nevertheless, genomic analysis seems to be more appropriate, as culturing failed to detect any *Fusobacterium* in the CRC resection material. Overall, the positive pilot results provide us with the confidence to move forward to a bigger cohort to overcome the significant limitations of a small sample group size and perform shotgun metagenomic sequencing on patient‐matched oral and CRC samples to map the associated microbiota in high resolution. This will provide the tool to identify whether specific oral strains of *Fusobacterium*, among others, are indeed associated with CRC and whether particular characteristics are enriched in these tumors.

## Author Contributions

**Niels Plomp:** analysis, interpretation, manuscript drafting. **Kristina Bertl:** idea, interpretation, sampling, manuscript editing. **Marie‐Louise Lydrup:** sampling, manuscript editing. **Klas Sjöberg:** design, interpretation, manuscript editing. **Hermie J.M. Harmsen:** design, interpretation, manuscript drafting, funding. **Andreas Stavropoulos:** idea, interpretation, sampling, funding, manuscript editing.

## Ethics Statement

The protocol was approved by the regional ethical review board (Dnr. 2016/469).

## Consent

All participants signed oral and written consent.

## Conflicts of Interest

The authors declare no conflicts of interest.

## Supporting information

Supplementary Table 1. Annotations of the amplicon sequence variants (ASV) of the CRC resection material obtained with 16S rRNA gene amplicon sequencing. The relative abundance of each ASV is shown per patient.

Supplementary Figure 1. Gel electrophoresis of the PCR products from *Fusobacterium* marker gene PCR of the patient saliva samples shows an amplicon of 610 bp in nine out of the ten patients. The PCR products were run together with a negative control (‐), a pure culture of *F. nucleatum* as a positive control (+), and the GeneRuler 100 bp plus DNA ladder (L) to estimate the amplicon size. Some patients are in duplicate, as multiple samples were collected for some of them. Samples marked with a cross (x) were excluded because no matching resection material was retrieved.

## Data Availability

The data that support the findings of this study are available from the corresponding author upon reasonable request.

## References

[cre270016-bib-0001] Abed, J. , N. Maalouf , A. L. Manson , et al. 2020. “Colon Cancer‐Associated *Fusobacterium nucleatum* May Originate From the Oral Cavity and Reach Colon Tumors via the Circulatory System.” Frontiers in Cellular and Infection Microbiology 10: 400. 10.3389/fcimb.2020.00400. 32850497 PMC7426652

[cre270016-bib-0002] Alexander, J. L. , J. M. Posma , A. Scott , et al. 2023. “Pathobionts in the Tumour Microbiota Predict Survival Following Resection for Colorectal Cancer.” Microbiome 11, no. 1: 100. 10.1186/s40168-023-01518-w. 37158960 PMC10165813

[cre270016-bib-0003] Almuzara, M. , C. Barberis , V. R. Velázquez , M. S. Ramirez , A. Famiglietti , and C. Vay . 2016. “Matrix‐Assisted Laser Desorption Ionization‐Time‐of‐Flight Mass Spectrometry (MALDI‐TOF MS) as a Reliable Tool to Identify Species of Catalase‐Negative Gram‐Positive Cocci Not Belonging to the *Streptococcus* Genus.” Open Microbiology Journal 10, no. December: 202–208. 10.2174/1874285801610010202. 28217192 PMC5278551

[cre270016-bib-0004] Bao, K. , X. Li , L. Poveda , et al. 2020. “Proteome and Microbiome Mapping of Human Gingival Tissue in Health and Disease.” Frontiers in Cellular and Infection Microbiology 10: 588155. 10.3389/fcimb.2020.588155. 33117738 PMC7566166

[cre270016-bib-0005] Bertl, K. , J. Burisch , N. Pandis , C. Bruckmann , B. Klinge , and A. Stavropoulos . 2022. “Periodontitis Prevalence in Patients With Ulcerative Colitis and Crohn's Disease—PPCC: A Case‐Control Study.” Journal of Clinical Periodontology 49, no. 12: 1262–1274. 10.1111/jcpe.13615. 35781889 PMC9804609

[cre270016-bib-0006] Borgnakke, W. S. 2019. “IDF Diabetes Atlas: Diabetes and Oral Health—A Two‐Way Relationship of Clinical Importance.” Diabetes Research and Clinical Practice 157, no. November: 107839. 10.1016/j.diabres.2019.107839. 31520714

[cre270016-bib-0007] Bullman, S. , C. S. Pedamallu , E. Sicinska , et al. 2017. “Analysis of Fusobacterium Persistence and Antibiotic Response in Colorectal Cancer.” Science 358, no. 6369: 1443–1448. 10.1126/science.aal5240. 29170280 PMC5823247

[cre270016-bib-0008] Casasanta, M. A. , C. C. Yoo , B. Udayasuryan , et al. 2020. “ *Fusobacterium nucleatum* Host‐Cell Binding and Invasion Induces IL‐8 and CXCL1 Secretion That Drives Colorectal Cancer Cell Migration.” Science Signaling 13, no. 641: eaba9157. 10.1126/scisignal.aba9157. 32694172 PMC7454160

[cre270016-bib-0009] Castellarin, M. , R. L. Warren , J. D. Freeman , et al. 2012. “ *Fusobacterium nucleatum* Infection Is Prevalent in Human Colorectal Carcinoma.” Genome Research 22, no. 2: 299–306. 10.1101/gr.126516.111. 22009989 PMC3266037

[cre270016-bib-0010] Chen, S. , T. Su , Y. Zhang , et al. 2020. “ *Fusobacterium nucleatum* Promotes Colorectal Cancer Metastasis by Modulating KRT7‐AS/KRT7.” Gut Microbes 11, no. 3: 511–525. 10.1080/19490976.2019.1695494. 31910722 PMC7524269

[cre270016-bib-0011] Cole, J. R. , Q. Wang , J. A. Fish , et al. 2014. “Ribosomal Database Project: Data and Tools for High Throughput rRNA Analysis.” Nucleic Acids Research 42, no. Database issue: D633–D642. 10.1093/nar/gkt1244. 24288368 PMC3965039

[cre270016-bib-0012] Domokos, Z. , E. Uhrin , B. Szabó , et al. 2022. “Patients With Inflammatory Bowel Disease Have a Higher Chance of Developing Periodontitis: A Systematic Review and Meta‐Analysis.” Frontiers in Medicine 9: 1020126. 10.3389/fmed.2022.1020126. 36425101 PMC9679143

[cre270016-bib-0013] Edgar, R. C. 2004. “MUSCLE: Multiple Sequence Alignment With High Accuracy and High Throughput.” Nucleic Acids Research 32, no. 5: 1792–1797. 10.1093/nar/gkh340. 15034147 PMC390337

[cre270016-bib-0014] Edgar, R. C. 2010. “Search and Clustering Orders of Magnitude Faster Than BLAST.” Bioinformatics 26, no. 19: 2460–2461. 10.1093/bioinformatics/btq461. 20709691

[cre270016-bib-0015] Felsenstein, J. 1981. “Evolutionary Trees From DNA Sequences: A Maximum Likelihood Approach.” Journal of Molecular Evolution 17, no. 6: 368–376. 10.1007/BF01734359. 7288891

[cre270016-bib-0016] Felsenstein, J. 1985. “Confidence Limits on Phylogenies: An Approach Using the Bootstrap.” Evolution 39, no. 4: 783–791. 10.1111/j.1558-5646.1985.tb00420.x. 28561359

[cre270016-bib-0017] Fu, M. M. , W. C. Chien , C. H. Chung , W. C. Lee , H. P. Tu , and E. Fu . 2022. “Is Periodontitis a Risk Factor of Benign or Malignant Colorectal Tumor? A Population‐Based Cohort Study.” Journal of Periodontal Research 57, no. 2: 284–293. 10.1111/jre.12955. 34854493

[cre270016-bib-0018] Gacesa, R. , A. Kurilshikov , A. Vich Vila , et al. 2022. “Environmental Factors Shaping the Gut Microbiome in a Dutch Population.” Nature 604, no. 7907: 732–739. 10.1038/s41586-022-04567-7. 35418674

[cre270016-bib-0019] Global Burden of Disease Collaborative Network . 2020. Global Burden of Disease Study 2019 (GBD 2019). Seattle, United States: Institute for Health Metrics and Evaluation (IHME).

[cre270016-bib-0020] de Goffau, M. C. , K. Luopajärvi , M. Knip , et al. 2013. “Fecal Microbiota Composition Differs Between Children With β‐Cell Autoimmunity and Those Without.” Diabetes 62, no. 4: 1238–1244. 10.2337/db12-0526. 23274889 PMC3609581

[cre270016-bib-0021] Graziani, F. , S. Gennai , A. Solini , and M. Petrini . 2018. “A Systematic Review and Meta‐Analysis of Epidemiologic Observational Evidence on the Effect of Periodontitis on Diabetes: An Update of the EFP‐AAP Review.” Journal of Clinical Periodontology 45, no. 2: 167–187. 10.1111/jcpe.12837. 29277926

[cre270016-bib-0022] Gur, C. , N. Maalouf , A. Shhadeh , et al. 2019. “ *Fusobacterium nucleatum* Supresses Anti‐Tumor Immunity by Activating CEACAM1.” OncoImmunology 8, no. 6: e1581531. 10.1080/2162402X.2019.1581531. 31069151 PMC6492956

[cre270016-bib-0023] Hajishengallis, G. 2022. “Interconnection of Periodontal Disease and Comorbidities: Evidence, Mechanisms, and Implications.” Periodontology 2000 89, no. 1: 9–18. 10.1111/prd.12430. 35244969 PMC9018559

[cre270016-bib-0024] Hajishengallis, G. , and T. Chavakis . 2021. “Local and Systemic Mechanisms Linking Periodontal Disease and Inflammatory Comorbidities.” Nature Reviews Immunology 21, no. 7: 426–440. 10.1038/s41577-020-00488-6. PMC7841384 33510490

[cre270016-bib-0025] Haririan, H. , O. Andrukhov , K. Bertl , et al. 2014. “Microbial Analysis of Subgingival Plaque Samples Compared to That of Whole Saliva in Patients With Periodontitis.” Journal of Periodontology 85, no. 6: 819–828. 10.1902/jop.2013.130306. 24144271

[cre270016-bib-0026] Heida, F. H. , A. G. J. F. van Zoonen , J. B. F. Hulscher , et al. 2016. “A Necrotizing Enterocolitis‐Associated Gut Microbiota Is Present in the Meconium: Results of a Prospective Study.” Clinical Infectious Diseases 62, no. 7: 863–870. 10.1093/cid/ciw016. 26787171

[cre270016-bib-0027] Kostic, A. D. , E. Chun , L. Robertson , et al. 2013. “ *Fusobacterium nucleatum* Potentiates Intestinal Tumorigenesis and Modulates the Tumor‐Immune Microenvironment.” Cell Host & Microbe 14, no. 2: 207–215. 10.1016/j.chom.2013.07.007. 23954159 PMC3772512

[cre270016-bib-0028] Kostic, A. D. , D. Gevers , C. S. Pedamallu , et al. 2012. “Genomic Analysis Identifies Association of *Fusobacterium* With Colorectal Carcinoma.” Genome Research 22, no. 2: 292–298. 10.1101/gr.126573.111. 22009990 PMC3266036

[cre270016-bib-0029] Kumar, S. , G. Stecher , M. Li , C. Knyaz , and K. Tamura . 2018. “MEGA X: Molecular Evolutionary Genetics Analysis Across Computing Platforms.” Molecular Biology and Evolution 35, no. 6: 1547–1549. 10.1093/molbev/msy096. 29722887 PMC5967553

[cre270016-bib-0030] Lamont, R. J. , H. Koo , and G. Hajishengallis . 2018. “The Oral Microbiota: Dynamic Communities and Host Interactions.” Nature Reviews Microbiology 16, no. 12: 745–759. 10.1038/s41579-018-0089-x. 30301974 PMC6278837

[cre270016-bib-0031] Li, W. , J. Xu , R. Zhang , et al. 2021. “Is Periodontal Disease a Risk Indicator for Colorectal Cancer? A Systematic Review and Meta‐Analysis.” Journal of Clinical Periodontology 48, no. 3: 336–347. 10.1111/jcpe.13402. 33179280

[cre270016-bib-0032] Lu, H. , P. Zou , Y. Zhang , Q. Zhang , Z. Chen , and F. Chen . 2022. “The Sampling Strategy of Oral Microbiome.” iMeta 1, no. 2: e23. 10.1002/imt2.23. 38868567 PMC10989882

[cre270016-bib-0033] Ma, X. , T. Sun , J. Zhou , et al. 2023. “Pangenomic Study of *Fusobacterium nucleatum* Reveals the Distribution of Pathogenic Genes and Functional Clusters at the Subspecies and Strain Levels.” Microbiology Spectrum 11, no. 3: e05184‐22. 10.1128/spectrum.05184-22. 37042769 PMC10269558

[cre270016-bib-0034] Madsen, G. R. , K. Bertl , N. Pandis , A. Stavropoulos , and J. Burisch . 2023. “The Impact of Periodontitis on Inflammatory Bowel Disease Activity.” Inflammatory Bowel Diseases 29, no. 3: 396–404. 10.1093/ibd/izac090. 35552410

[cre270016-bib-0035] Marotz, C. , J. T. Morton , P. Navarro , et al. 2021. “Quantifying Live Microbial Load in Human Saliva Samples Over Time Reveals Stable Composition and Dynamic Load.” mSystems 6, no. 1: e01182‐20. 10.1128/mSystems.01182-20. 33594005 PMC8561659

[cre270016-bib-0036] Marotz, C. A. , J. G. Sanders , C. Zuniga , L. S. Zaramela , R. Knight , and K. Zengler . 2018. “Improving Saliva Shotgun Metagenomics by Chemical Host DNA Depletion.” Microbiome 6, no. 1: 42. 10.1186/s40168-018-0426-3. 29482639 PMC5827986

[cre270016-bib-0037] Mima, K. , R. Nishihara , Z. R. Qian , et al. 2016. “ *Fusobacterium nucleatum* in Colorectal Carcinoma Tissue and Patient Prognosis.” Gut 65, no. 12: 1973–1980. 10.1136/gutjnl-2015-310101. 26311717 PMC4769120

[cre270016-bib-0038] Motoc, G. V. , R. I. Juncar , A. E. Moca , O. Motoc , L. Vaida , and M. Juncar . 2023. “The Relationship Between Age, Gender, BMI, Diet, Salivary pH and Periodontal Pathogenic Bacteria in Children and Adolescents: A Cross‐Sectional Study.” Biomedicines 11, no. 9: 2374. 10.3390/biomedicines11092374. 37760818 PMC10525996

[cre270016-bib-0039] Nagano, Y. , M. Watabe , K. G. Porter , et al. 2007. “Development of a Genus‐Specific PCR Assay for the Molecular Detection, Confirmation and Identification of *Fusobacterium* Spp.” British Journal of Biomedical Science 64, no. 2: 74–77. 10.1080/09674845.2007.11732760. 17633142

[cre270016-bib-0040] Peres, M. A. , L. M. D. Macpherson , R. J. Weyant , et al. 2019. “Oral Diseases: A Global Public Health Challenge.” Lancet 394, no. 10194: 249–260. 10.1016/S0140-6736(19)31146-8. 31327369

[cre270016-bib-0041] Richardson, M. , J. Ren , M. R. Rubinstein , et al. 2020. “Analysis of 16S rRNA Genes Reveals Reduced Fusobacterial Community Diversity When Translocating From Saliva to GI Sites.” Gut Microbes 12, no. 1: 1814120. 10.1080/19490976.2020.1814120. 33054632 PMC7577115

[cre270016-bib-0042] Rodríguez‐Lozano, B. , J. González‐Febles , J. L. Garnier‐Rodríguez , et al. 2019. “Association Between Severity of Periodontitis and Clinical Activity in Rheumatoid Arthritis Patients: A Case‐Control Study.” Arthritis Research & Therapy 21, no. 1: 27. 10.1186/s13075-019-1808-z. 30658685 PMC6339403

[cre270016-bib-0043] Rognes, T. , T. Flouri , B. Nichols , C. Quince , and F. Mahé . 2016. “VSEARCH: A Versatile Open Source Tool for Metagenomics.” PeerJ 4, no. October: e2584. 10.7717/peerj.2584. 27781170 PMC5075697

[cre270016-bib-0044] Rubinstein, M. R. , J. E. Baik , S. M. Lagana , et al. 2019. “ *Fusobacterium nucleatum* Promotes Colorectal Cancer by Inducing Wnt/β‐Catenin Modulator Annexin A1.” EMBO Reports 20, no. 4: e47638. 10.15252/embr.201847638. 30833345 PMC6446206

[cre270016-bib-0045] Sanz, M. , A. Marco del Castillo , S. Jepsen , et al. 2020. “Periodontitis and Cardiovascular Diseases: Consensus Report.” Journal of Clinical Periodontology 47, no. 3: 268–288. 10.1111/jcpe.13189. 32011025 PMC7027895

[cre270016-bib-0046] Shimomura, Y. , Y. Sugi , A. Kume , et al. 2023. “Strain‐Level Detection of *Fusobacterium nucleatum* in Colorectal Cancer Specimens by Targeting the CRISPR‐Cas Region.” Microbiology Spectrum 11 (October): e0512322. 10.1128/spectrum.05123-22. 37819098 PMC10714804

[cre270016-bib-0047] Socransky, S. S. , A. D. Haffajee , M. A. Cugini , C. Smith , and R. L. Kent . 1998. “Microbial Complexes in Subgingival Plaque.” Journal of Clinical Periodontology 25, no. 2: 134–144. 10.1111/j.1600-051x.1998.tb02419.x. 9495612

[cre270016-bib-0048] Sung, H. , J. Ferlay , R. L. Siegel , et al. 2021. “Global Cancer Statistics 2020: GLOBOCAN Estimates of Incidence and Mortality Worldwide for 36 Cancers in 185 Countries.” CA: A Cancer Journal for Clinicians 71, no. 3: 209–249. 10.3322/caac.21660. 33538338

[cre270016-bib-0049] Tamura, K. , and M. Nei . 1993. “Estimation of the Number of Nucleotide Substitutions in the Control Region of Mitochondrial DNA in Humans and Chimpanzees.” Molecular Biology and Evolution 10, no. 3: 512–526. 10.1093/oxfordjournals.molbev.a040023. 8336541

[cre270016-bib-0050] Tonetti, M. S. , H. Greenwell , and K. S. Kornman . 2018. “Staging and Grading of Periodontitis: Framework and Proposal of a New Classification and Case Definition.” Journal of Clinical Periodontology, Supplement, 45, no. S20 (June): 149. 10.1111/jcpe.12945. 29926495

[cre270016-bib-0051] Turnbaugh, P. J. , R. E. Ley , M. Hamady , C. M. Fraser‐Liggett , R. Knight , and J. I. Gordon . 2007. “The Human Microbiome Project.” Nature 449, no. 7164: 804–810. 10.1038/nature06244. 17943116 PMC3709439

[cre270016-bib-0052] Veloo, A. C. M. , E. D. de Vries , H. Jean‐Pierre , et al. 2016. “The Optimization and Validation of the Biotyper MALDI‐TOF MS Database for the Identification of Gram‐Positive Anaerobic Cocci.” Clinical Microbiology and Infection 22, no. 9: 793–798. 10.1016/j.cmi.2016.06.016. 27404365

[cre270016-bib-0053] Wang, Q. , G. M. Garrity , J. M. Tiedje , and J. R. Cole . 2007. “Naïve Bayesian Classifier for Rapid Assignment of rRNA Sequences Into the New Bacterial Taxonomy.” Applied and Environmental Microbiology 73, no. 16: 5261–5267. 10.1128/AEM.00062-07. 17586664 PMC1950982

[cre270016-bib-0054] Ximénez‐Fyvie, L. A. , A. D. Haffajee , and S. S. Socransky . 2000. “Microbial Composition of Supra‐ and Subgingival Plaque in Subjects With Adult Periodontitis.” Journal of Clinical Periodontology 27, no. 10: 722–732. 10.1034/j.1600-051x.2000.027010722.x. 11034118

[cre270016-bib-0055] Yang, Y. , W. Weng , J. Peng , et al. 2017. “ *Fusobacterium nucleatum* Increases Proliferation of Colorectal Cancer Cells and Tumor Development in Mice by Activating Toll‐Like Receptor 4 Signaling to Nuclear Factor‐κB, and Up‐Regulating Expression of MicroRNA‐21.” Gastroenterology 152, no. 4: 851–866.e24. 10.1053/j.gastro.2016.11.018. 27876571 PMC5555435

[cre270016-bib-0056] Yu, M. R. , H. J. Kim , and H. R. Park . 2020. “ *Fusobacterium nucleatum* Accelerates the Progression of Colitis‐Associated Colorectal Cancer by Promoting EMT.” Cancers 12, no. 10: 2728. 10.3390/cancers12102728. 32977534 PMC7598280

[cre270016-bib-0057] Yu, T. , F. Guo , Y. Yu , et al. 2017. “ *Fusobacterium nucleatum* Promotes Chemoresistance to Colorectal Cancer by Modulating Autophagy.” Cell 170, no. 3: 548–563.e16. 10.1016/j.cell.2017.07.008. 28753429 PMC5767127

[cre270016-bib-0058] Zhang, S. , Y. Yang , W. Weng , et al. 2019. “ *Fusobacterium nucleatum* Promotes Chemoresistance to 5‐Fluorouracil by Upregulation of BIRC3 Expression in Colorectal Cancer.” Journal of Experimental & Clinical Cancer Research: CR 38, no. 1: 14. 10.1186/s13046-018-0985-y. 30630498 PMC6327560

[cre270016-bib-0059] Zhernakova, A. , A. Kurilshikov , M. J. Bonder , et al. 2016. “Population‐Based Metagenomics Analysis Reveals Markers for Gut Microbiome Composition and Diversity.” Science 352, no. 6285: 565–569. 10.1126/science.aad3369. 27126040 PMC5240844

[cre270016-bib-0060] Zijnge, V. , T. Ammann , T. Thurnheer , and R. Gmür . 2012. “Subgingival Biofilm Structure.” Frontiers of Oral Biology 15: 1–16. 10.1159/000329667. 22142954

[cre270016-bib-0061] Zijnge, V. , M. B. M. van Leeuwen , J. E. Degener , et al. 2010. “Oral Biofilm Architecture on Natural Teeth.” PLoS One 5, no. 2: e9321. 10.1371/journal.pone.0009321. 20195365 PMC2827546

