# Supplementary material for: Does Fusobacterium in Colorectal Cancer Sites Originate From the Oral Cavity? A Pilot Study
Source: Clin Exp Dent Res. 2024 Nov 3;10(6):e70016. doi: 10.1002/cre2.70016 (PMC11532368; doi:10.1002/cre2.70016)
Supplement: Supplementary file 2 — Supplementary Figure 1. Gel electrophoresis of the PCR products from Fusobacterium marker gene PCR of the patient saliva samples shows an amplicon of 610 bp in nine out of the ten patients. The PCR products were run together with a negative control (‐), a pure culture of F. nucleatum as a positive control (+), and the GeneRuler 100 bp plus DNA ladder (L) to estimate the amplicon size. Some patients are in duplicate, as multiple samples were collected for some of them. Samples marked with a cross (x) were excluded because no matching resection material was retrieved. [file CRE2-10-e70016-s001.docx]

Supplementart Fig 1
